# Longitudinal associations between diurnal cortisol variation and later-life cognitive impairment

**DOI:** 10.1212/WNL.0000000000008729

**Published:** 2020-01-14

**Authors:** Alex Tsui, Marcus Richards, Archana Singh-Manoux, Chinedu Udeh-Momoh, Daniel Davis

**Affiliations:** From the MRC Unit for Lifelong Health and Ageing at UCL (A.T., M.R., D.D.) and Department of Epidemiology and Public Health (A.S.-M.), University College London, UK; Epidemiology of Ageing & Neurodegenerative Diseases (A.S.-M.), INSERM, U1153, Hotel Dieu, Paris, France; Neuroepidemiology and Ageing Research Unit (C.U.-M.), School of Public Health, Faculty of Medicine, The Imperial College of Science, Technology and Medicine, London; and Translational Health Sciences (C.U.-M.), Bristol Medical School, University of Bristol, UK.

## Abstract

**Objective:**

To determine whether hypothalamus-pituitary-adrenal axis (HPAA) dysfunction is prospectively associated with global cognitive impairment in later life.

**Methods:**

This cross-cohort study integrates 2 large longitudinal datasets, Whitehall II and the National Survey for Health and Development (NSHD), on data collected in the Whitehall II study between 2002–2004, 2007–2009, and 2012–2013; and for NSHD between 2006–2010 and in 2015. Serial salivary cortisol samples were collected multiple times within a 24-hour period at mean ages 61.2 and 65.9 years in Whitehall II and at age 60–64 years from NSHD participants. Cortisol profile is defined using cortisol awakening response and am:pm ratio. Cognitive function was measured using the Mini-Mental State Examination in Whitehall II and Addenbrooke’s Cognitive Examination, third version, in NSHD, harmonized into a 30-point score. Models were adjusted for age, sex, diagnoses of hypertension and diabetes, body mass index (BMI), educational attainment, and interval between HPAA and cognitive assessments.

**Results:**

In fully adjusted models, increased am:pm cortisol ratio was prospectively associated with better later-life cognitive function years later (0.02 fewer errors per SD increase in am:pm cortisol ratio, *p* < 0.01) and verbal fluency (0.03 SD increase in verbal fluency per SD increase in am:pm ratio, *p* < 0.01). Increasing age, lower educational attainment, diagnosis of hypertension, diagnosis of diabetes, and increased BMI were associated with worse cognitive function and poorer verbal fluency. There were no associations between depression and later-life cognition or reverse associations between cognition and later-life cortisol profiles.

**Conclusions:**

Loss of diurnal HPAA variation is evident in individuals subsequently experiencing more cognitive impairment. It may serve as an early preclinical marker of cognitive decline.

The hypothalamus-pituitary-adrenal axis (HPAA) is a major component of the physiologic stress response and known to mediate adaptive changes in brain function, notably memory, learning, and mood.^1^ Various strands of evidence have implicated the HPAA in the development and progression of cognitive impairment, though the precise nature of this relationship is unclear and may be bidirectional: the hypothalamus is regulated though projections from the hippocampus and the limbic system^2^—exactly those areas vulnerable to cognitive impairment common in preclinical or early Alzheimer pathology.

Individuals with neurodegeneration can demonstrate higher baseline plasma^3^ and CSF cortisol,^4^ as well as prolonged elevation of cortisol after an acute phase response.^5,6^ Cross-sectional neuroimaging studies have reported HPAA dysfunction to be associated with smaller hippocampal^7–9^ and reduced global brain volumes.^10^ Yet some longitudinal studies have found no association between cognition and peak cortisol^11^ or diurnal variation.^12^

Different cortisol measures are thought to have specific biological significance: for example, cortisol awakening response (CAR) has been associated with spatial memory and anticipation of cognitive demands,^13^ while blunted diurnal variation has been linked to reduced synaptic plasticity^14^ and hypothesized to be a global negative health profile marker.^15^ However, few population studies have profiled diurnal HPAA activity due to challenges of collecting serial cortisol measurements over 24 hours. Using serial measures of salivary cortisol in 2 population studies, we set out to answer the following question: Is HPAA dysfunction longitudinally associated with later-life global cognitive impairment and if so, is one cortisol measure specifically associated with cognitive impairment at follow-up?

## Methods

### Cohorts

**Figure 1:**
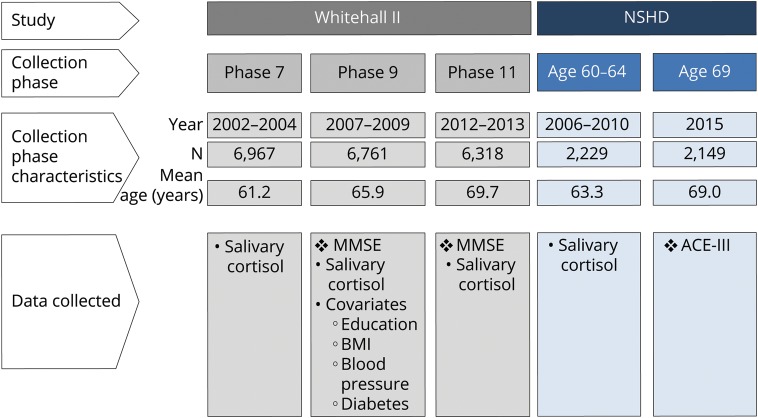
Data collection from Whitehall II and National Survey for Health and Development (NSHD) ACE-III = Addenbrooke's Cognitive Examination, third version; BMI = body mass index; MMSE = Mini-Mental State Examination.

This analysis integrates datasets from 2 longitudinal cohorts: the Whitehall II and National Survey for Health and Development (NSHD) studies (figure 1). The Whitehall II study is an ongoing study of men and women, originally employed by the British civil service, aged between 35 and 55 years and based in its London office (6,895 men and 3,413 women, response rate 73%) at recruitment to the study in 1985–1988.^16^ Since the initial medical examination, follow-up examinations have continued approximately every 5 years, with each wave taking 2 years to complete. This study obtained Whitehall II data from 2002–2004 (n = 6,967), 2007–2009 (n = 6,761), and 2012–2013 (n = 6,318).

The NSHD is the oldest British birth cohort study, following a sample of 5,362 male and female participants born in 1 week in March 1946.^17^ Between 2006 and 2010, when study members were aged between 60 and 64 years, 2,229 participants out of the 2,856 invited (78%) underwent clinical assessment at a clinical research facility (n = 1,690) or their own homes during a visit by a research nurse (n = 539). Of the remaining original participants, 778 had died, 570 were living abroad, 594 had previously withdrawn from the study, and 564 were lost to follow-up.

### Cognitive assessments

In Whitehall II, the Mini-Mental State Examination (MMSE) was administered at the 2007–2009 (mean age 65.9 years)^18^ and 2012–2013 (mean age 69.7 years)^19^ waves of data collection. In addition, participants were asked to generate in writing as many words beginning with S (phonemic fluency) and as many animal names (semantic fluency) as they could. One minute was allowed for each test; the observed range on these tests was 0–35.

In NSHD, when participants were 69 years of age, cognition was assessed in 2,149 participants using Addenbrooke’s Cognitive Examination, third version (ACE-III), with a total score of 100, divided into 5 domains: attention and orientation (scored 0–18), verbal fluency (0–14), memory (0–26), language (0–26), and visuospatial function (0–16). A customized version of the ACE-III was administered by iPad using ACE Mobile (acemobile.org/): 32 refused or were unable to undertake the ACE-III; 35 did not have fully completed ACE-III scores; 353 scores were lost through equipment failure. Complete ACE-III data were available for 1,729 participants (80.5% of those who received home visit).^20^ From the ACE-III score, a 30-point scale directly comparable to the MMSE score was extracted in order to harmonize cognitive measures with the Whitehall II study. In addition, verbal memory and visual search speed were assessed earlier, when participants were 53 years old; these were taken to be measures of baseline cognitive function prior to the wave first measuring cortisol (see below). While phonemic fluency was assessed in writing by Whitehall II, participants in NSHD were verbally asked to name the words aloud. The 2 fluency scores were then standardized across individual cohort distributions. The raw scores and distributions are available online for each wave of both Whitehall II and NSHD (table e-1, doi.org/10.5061/dryad.vb3g6p1).

### Cortisol measures

Salivary cortisol samples were obtained at a mean age of 61.2 and 65.9 years in Whitehall II and at age 60–64 years from NSHD participants (figure 1). Prior to the sample collection, participants were asked to avoid brushing or flossing their teeth and eating, drinking, or smoking for 30 minutes before. Salivary cortisol was collected using a Salivette swab. Whitehall II participants were asked to provide a total of 6 saliva samples: on waking; at 30 minutes, 2.5 hours, 8 hours, and 12 hours after waking; and at bedtime. NSHD participants were asked to provide 4 saliva samples within 24 hours: during their clinic appointment, in the subsequent evening between 9 pm and 9:30 pm on the same day as their clinic appointment, on waking the following day, and 30 minutes after waking. Participants were asked to refrigerate the swab before posting the sample to the laboratory in a protected container. A booklet was used for participants to record information on the day of sampling including date of collection, wake time, and time each sample was taken. Salivary cortisol levels were measured using a commercial immunoassay with chemiluminescence detection (CLIA; IBL-Hamburg, Germany). The limit of detection was 0.44 nmol/L; intra-assay and interassay coefficients of variance were less than 8%. Any sample greater than 50 nmol/L was repeated. Consistent with previous analyses, anomalous salivary profiles were excluded^21,22^: these included use of corticosteroid medications, timing of collections suggestive of shift work patterns (morning sample taken before 4 am or after midday; evening sample taken before 8 pm), lack of sample collection timings, incorrect chronological order of sample timings, or any cortisol concentration more than 3 SD from the mean.

### Covariates

Hypertension was defined as a diagnosis of hypertension, regular prescription of an antihypertensive, or systolic blood pressure greater than 140 mm Hg or diastolic blood pressure greater than 90 mm Hg (taken from 2 readings). Diabetes was defined as participant-reported doctor-diagnosed type 1 or 2 diabetes mellitus, fasting glucose 7.0 mmol/L, a 2-hour post-load glucose 11.1 mmol/L, or use of diabetes medication. Height and weight were measured by standardized protocols, and body mass index (BMI) was calculated (kg/m^2^). Educational attainment was defined as highest qualification on leaving full-time education in Whitehall II participants and by age 26 in NSHD participants. Depression caseness was defined using the General Health Questionnaire to ascertain depression symptoms, as previous operationalized by Goldberg and Hillier^23^ and within the NSHD cohort by James et al.^24^: scores of 5 or over (with the Likert scale recoded to 0-0-1-1 for each item before summing) were used as the threshold. For NSHD, covariates were taken from data collection at 60–64 years, the only wave with available cortisol measures available. For Whitehall II, covariates were taken from the data collection wave with the second cortisol assessment (2007–2009), when the 2 cohorts were at comparable ages, and any missing covariate data were updated from the earlier cortisol assessment if available.

### Statistical analysis

Global cognitive state was measured on a 30-point scale (MMSE in Whitehall II; comparable scale extracted from 100-point ACE-III in NSHD). Given that the distribution of the MMSE is negatively skewed, it is often transformed using log(31 − MMSE score).^25^ Two cortisol measures were derived: (1) CAR, calculated by peak cortisol minus cortisol on waking; (2) diurnal variation as defined by am:pm ratio, calculated by the peak cortisol divided by the sample taken latest in the day (sample 6 in Whitehall II, sample 4 in NSHD). In order to compare exposures directly, continuous measures (verbal fluency scores, CAR, log-transformed am:pm ratios) were standardized to have a mean of 0 and SD of 1. Covariates were operationalized as hypertension (yes/no), diabetes (yes/no), BMI (continuous), and educational attainment (<O levels, O levels, A levels).

Linear regression was used to estimate the longitudinal association of cortisol measures with later-life cognitive performance, years after the cortisol collection. The model was lagged such that the cortisol measure in the model was from one data collection wave prior to the cognitive measure and all estimates were adjusted by follow-up interval. Covariates used were from the 2007–2009 wave of Whitehall II and ages 60–64 of NSHD using complete cases. Models were also estimated using multiple imputation for missing covariates (20 datasets obtained through chained equations using the mi impute command in Stata). Univariate analyses were first performed between cognition with each cortisol measure and covariate, followed by a fully adjusted model. All data collected from Whitehall II from the same participant over multiple waves were clustered by participant. Finally, the same models were repeated with reversed outcomes and exposures, with cortisol measures as outcomes and cognitive measures as lagged exposures. Stata version 14.1 (StataCorp, College Station, TX) was used for all analyses.

### Data availability

Bona fide researchers can apply to access the Whitehall II data and NSHD data via a standard application procedure. Deidentified patient data are available for sharing with any other bona fide researcher for high-quality research output. Individual level and aggregate data are available from Whitehall II across 12 phases of data collection beginning in 1985. Aggregate data are available for NSHD across 24 waves of data collection beginning in 1946. All data sharing must be within the bounds of consent given previously by study members and meet rigorous data security standards, adhering to the core principles of ethical, equitable, and efficient data sharing as set out by the Medical Research Council (UK) and subject to a data sharing agreement. Applications for data sharing can be made via established protocols as outlined by Medical Research Council Unit of Lifelong Health and Ageing at UCL (nshd.mrc.ac.uk/data/data-sharing/) and Whitehall II (ucl.ac.uk/iehc/research/epidemiology-public-health/research/whitehallII/data-sharing).

## Results

The mean ages of participants from the 3 waves of Whitehall data at 2002–2004, 2007–2009, and 2012–2013 were 61, 65.8, and 63.3 years, respectively (table 1). Women in both cohorts demonstrated a greater CAR; men demonstrated a larger am:pm ratio. Transformation of the MMSE errors normalized the distribution to be sufficiently appropriate for the analysis (mean 0.87, median 0.69, kurtosis 2.62, skewness 0.08; figure e-1, doi.org/10.5061/dryad.vb3g6p1). Greater educational attainment was obtained by men in both cohorts while hypertension and greater BMI was more prevalent in men in the Whitehall II cohort. The mean and SD for each cognitive outcome and cortisol measure and prevalence are described in table 1. The interaction term between am:pm ratio and sex did not reveal sex between MMSE errors and am:pm ratio (*p* = 0.770), CAR (*p* = 0.902), or area under the curve (AUC) (*p* = 0.455), or between fluency and am:pm ratio (*p* = 0.411), CAR (*p* = 0.880), or AUC (*p* = 0.223).

**Table 1:**
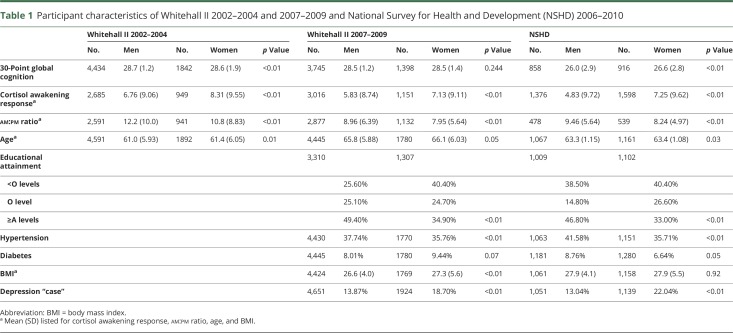
Participant characteristics of Whitehall II 2002–2004 and 2007–2009 and National Survey for Health and Development (NSHD) 2006–2010

There was an association between am:pm ratio and subsequent cognitive function in univariate (table 2) analyses (0.03 fewer MMSE errors per SD increase in am:pm ratio per year, 95% CI 0.02 errors to 0.04 errors, *p* < 0.01), which remained when fully adjusted (0.02 fewer MMSE errors per SD increase in am:pm ratio, 95% CI 0.00–0.03, *p* = 0.01) (table 3). An association between am:pm ratio and verbal fluency was also evident (0.06 SD increase in verbal fluency per SD increase in am:pm ratio, 95% CI 0.04–0.08, *p* < 0.01), which remained on full adjustment (0.03 SD increase in verbal fluency per SD increase in am:pm ratio, 95% CI 0.01–0.05, *p* < 0.01). Increasing age, male sex, greater educational attainment, increased BMI, and diagnoses of hypertension and diabetes were also associated with poorer later-life cognitive function. An association between depression caseness and later-life cognition attenuated on full adjustment (95% CI −0.05 to 0.01, *p* = 0.17).

**Table 2:**
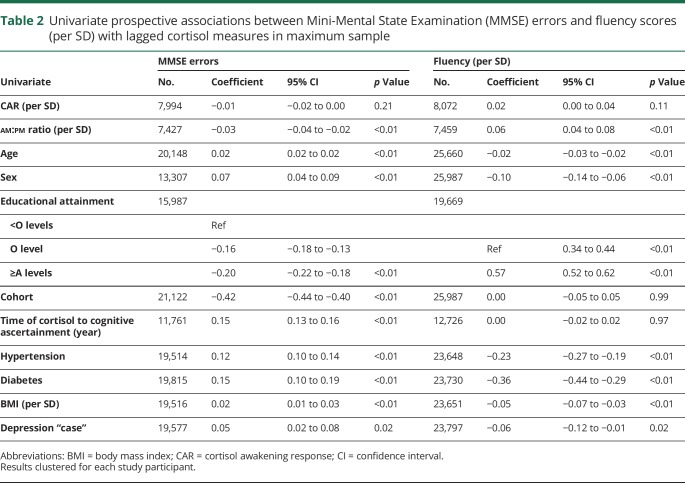
Univariate prospective associations between Mini-Mental State Examination (MMSE) errors and fluency scores (per SD) with lagged cortisol measures in maximum sample

**Table 3:**
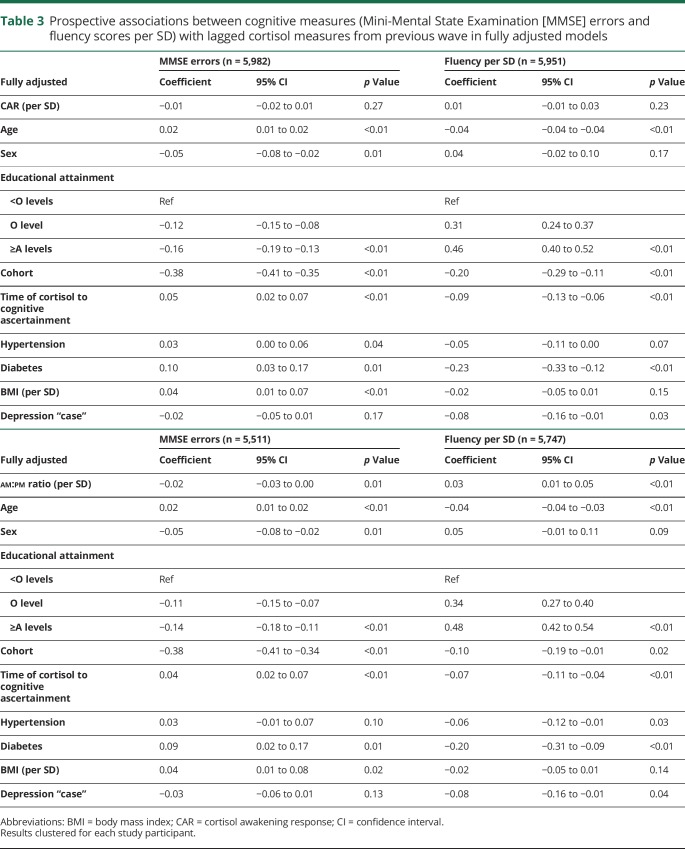
Prospective associations between cognitive measures (Mini-Mental State Examination [MMSE] errors and fluency scores per SD) with lagged cortisol measures from previous wave in fully adjusted models

No associations were found between CAR and subsequent MMSE errors or verbal fluency in univariate or fully adjusted models. Increasing age, lower educational attainment, diagnosis of hypertension, diagnosis of diabetes, increased BMI, and increased delay between cortisol measurement and cognitive assessment were all associated with worse cognitive function (table 3). Each was also associated with poorer verbal fluency (except follow-up interval). Overall, no prospective associations between cognitive function and subsequent cortisol were evident.

Finally, in considering contribution of baseline cognitive function, corresponding and directly comparable cognitive assessments at baseline was only available for the Whitehall II participants (table e-4, doi.org/10.5061/dryad.vb3g6p1). In further multivariate analyses within the Whitehall subset, the prospective association between cortisol diurnal variation and later-life cognitive performance persisted even when baseline cognitive performance was adjusted for (table e-4, doi.org/10.5061/dryad.vb3g6p1). For verbal fluency, the association was attenuated on adjustment for performance in the prior data collection wave (table e-4, doi.org/10.5061/dryad.vb3g6p1). Between measures of cortisol within each analysis, approximately 69% of the harmonized cohorts had complete measures of cortisol measures, later-life cognitive outcomes, and all covariates. All models were repeated using multiple imputation data to account for missing covariates, the findings of which did not differ from the main analyses (table e-3, doi.org/10.5061/dryad.vb3g6p1).

## Discussion

In our longitudinal cross-cohort study, decreased am:pm cortisol ratio was prospectively associated with small impairments in later-life cognition and subsequently decreased verbal fluency following adjustment for age, sex, educational attainment, hypertension, diabetes, BMI, and depression. No prospective association was found between CAR and cognitive function at follow-up. No reverse associations were found between cognition and later-life cortisol measures. Taken together, these findings suggest blunted HPAA diurnal variation precedes and may contribute to increased risk of later-life cognitive decline in the general population.

These results should be interpreted in the context of strengths and weaknesses of the study design. A main strength was the large sample size made possible by integration of 2 longitudinal cohorts with harmonized exposures, outcomes, and a long history of collaboration, harmonization, and participation in consortia.^26^ Second, the 5- and 9-year period from cortisol measurement to cognitive assessment allowed longer-term outcomes to be ascertained. Multiple cortisol sample collections over 24 hours allowed for accurate profiling of HPAA activity. Finally, cortisol collection from saliva instead of serum^11^ minimized acute stress response caused by venipuncture while measuring free, unbound cortisol.

Nonetheless, challenges in cortisol collection were evident from the amount of missing data in both cohorts, and this is likely to be missing-not-at-random. For example, individuals with lower cognitive measures are less likely to have complete 24-hour cortisol profiles, introducing potential source of selection bias in our complete case approach. However, estimating these models again using multiple imputation did not produce different conclusions (table e-3, doi.org/10.5061/dryad.vb3g6p1). In addition, while the 2 cohorts are comparable in most respects, the exact collection schedule for salivary cortisol differed (6 times/24 hours in Whitehall II; 4 times/24 hours in NSHD). As a result, while AUC would have been the optimal method to characterize cortisol profiling, AUC calculation would have been more accurate for Whitehall II compared to NSHD. am:pm ratio was chosen as a measure of diurnal blunting instead to utilize 2 directly comparable collection points. In addition, cohort-specific estimates were affected through differences in data collection. Baseline cognition was only available in Whitehall II participants, while other specific cognitive domains were only repeated in NSHD participants. An association between diurnal variation and later-life cognition was still evident in Whitehall II participants adjusting for baseline cognition, and coefficient directions for search speed and verbal memory were consistent (though underpowered) in NSHD participants (tables e-6 and e-7, doi.org/10.5061/dryad.vb3g6p1). In common with other cohort studies, our findings may be subject to residual confounding and are specific to the population and era under study. Finally, translation of our findings into diagnoses of dementia was not possible due to the relatively low number of clinical cases in this age range. However, cognitive impairment as demonstrated by MMSE and ACE-III in Whitehall II and NSHD were uncommon at the ages we examined: while not a definition for dementia, MMSE scores under 24 were found in only 0.48%, 0.78%, and 1.84% for Whitehall II waves 7, 9, and 11, respectively; and ACE-III scores of under 82 in 6.5% of NSHD participants at age 69.

While there are relatively few studies of similar size for comparison, our finding that HPAA dysfunction is associated with later-life cognitive impairment is in keeping with findings of the Baltimore Longitudinal Study of Aging and the Longitudinal Aging Study of Amsterdam, which reported increased diurnal variation and random cortisol levels in similarly aged older adults to be associated prospectively with impaired cognitive function and Alzheimer disease risk, respectively.^11,27^ In addition, HPAA dysfunction had also been previously associated with prospective cognitive decline in cognitively healthy adults with positive β-amyloid on PET scans, suggesting that HPAA dysfunction may be a marker of, share, or even mediate pathogenic β-amyloid mechanisms in cognitive impairment that may represent early preclinical phases of Alzheimer disease.^28^ Our findings also suggest an association between HPAA dysfunction and cognition only becomes evident at older ages,^27^ in keeping with a sensitive period for cognitive decline.^29^ Similar associations were not present at younger ages from both constituent cohorts: there were no prospective associations between cortisol profiles at 2002–2004 and cognitive assessments at 2007–2009 in Whitehall II^12^; there were no cross-sectional associations between cortisol diurnal variation and cognitive measures at ages 60–64 in NSHD participants.^22^

The specific association of am:pm ratio with later-life cognition highlights the significance of blunted HPAA diurnal variation. Although the demonstrated effect sizes appear small, even small per year individual effects may have cumulative effect when considered at a population level, particularly when cognitive decline may subclinically progress over decades. Second, the finding suggests an additional mechanism apparently contributing to cognitive aging in the population, above more established risk factors such as diabetes and hypertension. This implicates HPAA dysfunction as a potential pathogenic factor towards later-life cognitive impairment, providing a possible novel target for future research and potential therapeutic targets. On a cellular level, blunted diurnal variation is associated with reduced synaptic plasticity.^14^ In addition, blunted diurnal variation is hypothesized to indicate reduced capacity to adapt during aging and changes in cardiovascular profile.^15,30^ Consequently, reactions from endocrine, immune, and cardiovascular systems may be initiated or amplified to environmental and psychosocial stressors, contributing to negative health consequences such as dementia.^31^ The lack of prospective association between CAR and cognition in our study may indicate a more nuanced role for CAR that is difficult to ascertain given data limitations within our study. Although associated with spatial memory,^32^ CAR is thought to anticipate cognitive demands by taking into account previous experiences,^13^ hence its absence in patients with retrograde amnesia,^33^ after waking from a nap,^34^ and when waking in the middle of the night.^35^ Such a role for CAR is anatomically plausible, given hippocampal inputs to the hypothalamus and the function of the hippocampus to provide a cohesive construct and representation of the outside world by processing relationships with time, space, and environmental cues.

Given that a blunted HPAA diurnal profile may provide a physiologic substrate for incipient cognitive decline in the population, HPAA dysfunction could offer a potential target for disease modification in dementia. Selective inhibition of 11-β-hydroxysteroid dehydrogenase-1, an enzyme selectively expressed in human hippocampus, cerebellum, and frontal cortex, that regenerates glucocorticoid from circulating cortisone, amplifying intracellular glucocorticoid concentrations, has been associated with improved verbal fluency in healthy older men, and verbal memory in type 2 diabetic older men.^36^ Overall, the relationship between HPAA and cognition is complex; nonetheless, our findings support loss of diurnal HPAA variation as an early marker of later-life cognitive impairment and a potential future target for disease modification.

## Glossary

ACE-III: Addenbrooke’s Cognitive Examination, third version
AUC: area under the curve
BMI: body mass index
CAR: cortisol awakening response
HPAA: hypothalamus-pituitary-adrenal axis
MMSE: Mini-Mental State Examination
NSHD: National Survey for Health and Development
